# Unilateral Upper Cervical Cord Infarction: A Report of Two Cases with Mild Neurological Symptoms Accompanying a Small Ischemic Lesion Detected by Brain MRI

**DOI:** 10.1155/2020/8836561

**Published:** 2020-10-05

**Authors:** Manabu Wada, Hikaru Nagasawa, Yoshitaka Yamaguchi

**Affiliations:** Department of Neurology, Yamagata Prefectural Central Hospital, 1800 Aoyagi, Yamagata, Japan

## Abstract

Spinal cord infarction (SCI) is rare, difficult to diagnose, and often fails to be detected by diffusion-weighted imaging (DWI) of spinal cord magnetic resonance imaging (MRI). Because the clinical features of SCI can vary widely, diagnosis during the acute phase of SCI is often challenging for clinicians. Although SCI shares similar etiologies with cerebral infarction, the characteristics of SCI without vessel dissection remain largely unknown. We present two older patients with mild neurological symptoms who each presented with a small, unilateral, upper cervical cord lesion, which was detected by thin-section, coronal DWI of brain MRI. Both unilateral small lesions were localized in the right lateral funiculus, and each patient showed good prognosis. The anatomical findings suggested that the pial collateral network surrounding the cervical cord contributed to lesion formation. Small and localized lesions have been associated with mild neurological symptoms and better short-term prognosis. The present report indicated that the use of thin-section coronal DWI when performing brain MRI may be helpful for the diagnosis of small, unilateral, upper cervical cord infarctions.

## 1. Introduction

Spinal cord infarction (SCI) is a rare disease that causes devastating neurological sequelae, such as paraplegia and incontinence [1–4]. SCI diagnosis during the acute phase is often challenging for clinicians because the clinical features of SCI can vary widely [1–4]. Numerous collateral systems, constructed by spinal cord vascular networks, may account for the rarity and clinical variations observed for SCI [1, 3, 4]. The most common etiologies of SCI include vascular risk factors, aortic disease, and vessel dissection; however, the mechanisms underlying spontaneous or nonsurgical SCI remain largely unclear [1, 3, 4].

A recent clinical study examining cervical cord infarctions in patients with and without vessel dissection showed that lesions were frequently located in the upper cervical cord among patients with dissection, whereas patients without dissection presented with an older mean age of onset and the lesions tended to be located in the lower cervical regions [2]. Owing to the rarity of SCI, the clinical characteristics and magnetic resonance imaging (MRI) findings associated with unilateral, upper cervical cord infarction without vascular dissection have been seldom discussed.

We present two cases of older patients with mild neurological symptoms and small, unilateral, upper cervical cord lesions that were detected by thin-section, coronal, diffusion-weighted imaging (DWI) of brain MRI.

## 2. Case Presentation

### 2.1. Case 1

**Figure 1:**
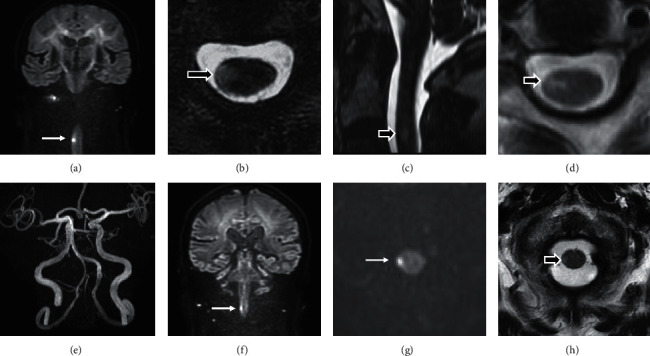
Magnetic resonance imaging (MRI) of spinal infarctions in two cases. The lesion for case 1 is presented in (a–e). (a) Thin-section coronal diffusion-weighted imaging (DWI), showing a high-intensity lesion of the cervical spine (arrow). (b and c) 3D fast imaging employing steady-state acquisition; MRI showing an upper cervical lesion. Axial (b) and sagittal (c); MRI showing a small, high-intensity lesion (arrows) at the level of the C2/3 intervertebral disc. (d) Axial, T2-weighted image of the cervical cord, examined 5 weeks after onset. (e) MR angiography of case 1, showing no apparent dissection of vertebral arteries. The MRI findings of case 2 are presented in (f–h). (f) Thin-section, coronal DWI and (g) axial DWI, demonstrating a high-intensity lesion (arrows) in the upper cervical cord. (h) T2-weighted axial image, showing a high-intensity lesion (arrow) in the right lateral funiculus at the level of the C1 vertebra.

An 84-year-old woman with a history of diabetes and hypertension experienced acute onset of severe right neck pain. Neurological examination showed no apparent abnormality. Brain MRI was performed, including thin-section, coronal DWI, and neither abnormal signal changes nor vertebral artery dissection were detected. Four days later, she noticed the impairment of temperature perception in her left foot while bathing. Subsequent neurological examination showed mild hypoalgesia of the left upper and lower limbs. No weakness, disturbance of vibration sense, or urinary incontinence was apparent. Brain MRI was performed again, and thin-section, coronal DWI showed a high-intensity lesion in the right lateral funiculus, within the cervical spinal cord, at the level of the C2/C3 intervertebral disc (Figure 1(a)). Subsequently, fast imaging employing steady-state acquisition sequences showed a small, hyperintense lesion in the right lateral funiculus (Figures 1(b) and 1(c)). Repeated magnetic resonance (MR) angiography showed mild atherosclerosis; however, no double-lumen signs, strings, or pearls signs were observed. Therefore, we concluded that the patient did not have any artery dissection (Figure 1(e)). She was able to walk without any assistance, but the temperature perception impairment in her limbs remained unchanged 4 weeks after onset. Follow-up MRI, examined 5 weeks after onset, showed a high-intensity lesion of the right lateral funiculus and anterior horn, within the cervical spinal cord (Figure 1(d)).

The settings used for the MRI protocols are summarized in the Table 1.

### 2.2. Case 2

A 74-year-old man with a history of prostate cancer, cholecystolithiasis, and hypertension experienced an unsteady gait 3 days after laparoscopic cholecystectomy. A neurological examination showed mild ataxia of his right lower limb, and tandem gait was impaired. No apparent weakness or sensory disturbance was found. Brain MRI was performed, and thin-section, coronal DWI showed a high-intensity lesion in the right lateral funiculus, within the cervical spinal cord, at the level of C1 (Figure 1(f)). Axial DWI showed a hyperintense lesion of the spinal cord (Figure 1(g)), and the corresponding T2-weighted images (Figure 1(h)) demonstrated a hyperintense area in the right lateral funiculus. MR angiography showed an occlusion in the right vertebral artery. Short-term prognosis was good, and he was able to walk without any assistance.

## 3. Discussion

Development of collateral circulation is associated with the rarity of SCI and variations in symptoms and lesion sizes: although the etiology of SCI is similar to that of cerebral infarction and systemic vascular disease, SCI is estimated to represent only 1% to 2% of all ischemic strokes [2, 5, 6]. The clinical features of SCI depend on the lesion location and the degree of arterial occlusion [3, 6]. Anatomically, the anterior two-thirds of the spinal cord and the anterior portion of the posterior column receive blood via the anterior spinal artery (ASA), whereas the posterior one-third of the spinal cord is supplied by the two posterior spinal arteries (PSAs) [7, 8]. These arteries construct collateral circulation networks, with individual variations, which may contribute to the rarity and clinical variations associated with the neurological symptoms that present in patients with SCI.

**Figure 2:**
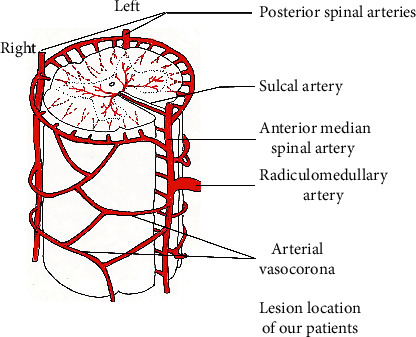
Schema showing the arterial supply to the cervical spinal cord (the figure is adapted from [9]).

Associations between mild neurological symptoms and the locations of small cervical cord lesions in our patients: anatomically, the vasocorona that covers the cervical cord contributed to the formation of localized, unilateral, small-sized infarcts in our patients (Figure 2). The MRI findings in our patients showed high-intensity lesions in the lateral funiculus of the cervical cord on DWI and T2-weighted imaging in each patient. The surface layer of the lateral funiculus is supplied primarily by the coronary artery, which arises from the ASA. The medial region includes the gray matter, and the region of the lateral funiculus where the lesions were localized is supplied by the central artery [10]. A potential watershed zone has been hypothesized to exist between the regions supplied by the ASA and PSA, involving parts of the corticospinal and spinothalamic tracts [11], although little evidence has been presented to confirm its existence. In our patients, the unilateral, small-sized lesions were limited to the lateral funiculus and were associated with mild symptoms. We hypothesize that injuries to the spinothalamic tract may have contributed to the experience of neck pain and mild limb ataxia in our patients. Previous studies have indicated that central pain is likely to occur after the impairment of the spinothalamic tract, which is located in the lateral funiculus [4, 12].

Detection of small infarctions in the upper cervical cord using thin-section, coronal DWI of brain MRI: unilateral and small-sized SCI is exceedingly rare, and MRI sometimes fails to detect small ischemic lesions of the spinal cord. Kumral and colleagues have previously reported that in patients with SCI, spinal MRI was effective in only a portion of patients with unilateral ischemic lesions (57% in their series) [1]. The failure of spinal cord MRI to detect cervical lesions may be related to the phase of the illness or the size of the SCI [7]. In contrast to the mild neurological symptoms presented by our patients, most of the patients described by Kumral and colleagues showed moderate-to-severe neurological disturbances and required walking assistance or a wheelchair upon leaving the hospital [1].

Although DWI is the current gold-standard for the detection of acute ischemic stroke [13], DWI sometimes fails to detect very small ischemic lesions, especially those in the posterior fossa. Recent studies have shown that the performance of additional thin-section, coronal DWI of the posterior fossa can overcome this problem. Coronal plane slices in the upper cervical cord may be positioned perpendicular to sections of the coronary arteries associated with the ASA and the pial collateral network, allowing the capture and identification of even very small ischemic lesions in our patients.

Clinicians should be aware that hyperintense lesions on DWI may also be observed in the spinal cord during acute stage inflammatory diseases. In particular, “clinically isolated syndrome” sometimes shows an isolated lesion that is located peripherally and contacts the surface of the spinal cord [3]. A previous study indicated the importance of measuring apparent diffusion coefficient (ADC) values, which were found to be lower in spinal cord infarcts than in inflammatory spinal cord lesions [14]. Therefore, the measurement of ADC values may be useful for differentiating SCI from inflammatory myelopathies during the acute stages of these diseases.

The present study indicated that thin-section, coronal DWI can detect the presence of an upper cervical cord infarction. However, the performance of coronal DWI for the detection of ischemic lesions in the upper cervical cord, relative to the performance of sagittal DWI, could not be evaluated. This is because we did not perform sagittal DWI during the spinal cord MRI in the acute phases of the disease in these patients.

## 4. Conclusion

We present two older patients, with unilateral, small-sized, cervical infarction, which were detected by thin-section, coronal DWI of brain MRI. Small and localized lesions may be related to good prognosis. Whether thin-section coronal DWI is superior to spinal cord sagittal DWI for the detection of upper cervical cord ischemic lesions remains uncertain. Further studies, using larger samples of patients with unilateral upper cervical cord infarctions, remain necessary to clarify the prognosis and advantages of thin-section, coronal DWI.

## Figures and Tables

**Table 1:** Summary of MRI protocols examined in our cases.

Parameters	Case 1				Case 2		
	DWI coronal	FIESTA axial	FIESTA sagittal	T2WI (spine)	DWI coronal	DWI axial	T2WI axial
Magnetic field strength	3.0 T				3.0 T		
Matrix size	288 × 288	512 × 512	1024 × 1024	512 × 512	282 × 282	320 × 320	704 × 704
Field of view	240 mm × 240 mm	160 mm × 160 mm	180 mm × 180 mm	180 mm × 180 mm	240 mm × 240 mm	240 mm × 240 mm	240 mm × 240 mm
Slice thickness (mm)	3	1.6	2	4	3	3	5
Repetition time (ms)	5000	1500	1500	3025	5000	5000	4000
Echo time (ms)	137	256	256	100	137	85	104
*b* values	1000				1000	1000	

DWI, diffusion-weighted image; FIESTA, 3D fast imaging employing steady-state acquisition; T2WI, T2-weighted image.

## Data Availability

The data used to support the study are available within the article.
